# Environmental tobacco smoke exposure among non-smoking waiters: measurement of expired carbon monoxide levels

**DOI:** 10.1590/S1516-31802000000400003

**Published:** 2000-07-07

**Authors:** Ronaldo Laranjeira, Sandra Pillon, John Dunn

**Keywords:** Passive Smoking, Restaurants, Carbon Monoxide, Brazil, Fumante passivo, Restaurantes, Monóxido de carbono, Brasil

## Abstract

**CONTEXT:**

Exposureto environmental tobacco smoke is a health risk that is of concern to patrons and of particular concern to employees of restaurants and bars.

**OBJECTIVE:**

To assess environmental tobacco smoke exposure (using expired carbon monoxide levels) in non-smoking waiters before and after a normal day's shift and to compare pre-exposure levels with non-smoking medical students.

**DESIGN:**

An observational study.

**SETTING:**

Restaurants with more than 50 tables or 100 places in São Paulo.

**SUBJECTS:**

100 non-smoking restaurant waiters and 100 non-smoking medical students in São Paulo, Brazil.

**MAIN MEASUREMENTS:**

Levels of expired carbon monoxide, measured with a Smokerlyser (Bedfont EC 50 Scientific), before and after a normal day's work.

**RESULTS:**

Waiters’ pre-exposure expired carbon monoxide levels were similar to those of medical students, but after a mean of 9 hours exposure in the workplace, median levels more than doubled (2.0 ppm vs. 5.0 ppm, P <0.001). Post-exposure carbon monoxide levels were correlated with the number of tables available for smokers (Kendall's tau = 0.2, P <0.0001).

**CONCLUSIONS:**

Exposure to environmental tobacco smoke is the most likely explanation for the increase in carbon monoxide levels among these non-smoking waiters. These findings can be used to inform the ongoing public health debate on passive smoking.

## RESUMO

**CONTEXTO:**

O ambiente exposto à fumaça do cigarro traz graves riscos à saúde e preocupa tanto os empregadores quanto empregados de restaurantes e bares. No entanto, os fumantes passivos correm riscos elevados de desenvolverem doenças cancerígenas, pulmonares e cardíacas. Baseados nestes parâmetros e na experiência de países que sancionaram leis que proíbem fumar ou restringem o fumar em áreas restritas e, antes de sancionar leis municipais na cidade de São Paulo, dá-se a importância de realizar este estudo.

**OBJETIVO:**

Avaliar a exposição à fumaça de cigarro no ambiente de trabalho (através da medida do nível de monóxido de carbono) em garçons não fumantes, antes e depois do expediente de trabalho, e comparar os níveis pré-exposição com os de estudantes universitários não fumantes.

**TIPO DE ESTUDO:**

Estudo observacional.

**LOCAL:**

Restaurantes com mais de 50 mesas ou 100 assentos, todos localizados em São Paulo.

**PARTICIPANTES:**

A amostra é constituída por 100 garçons não fumantes e 100 estudantes não fumantes.

**VARIÁVEIS ESTUDADAS:**

O nível de monóxido de carbono expirado foi medido com um aparelho Smokerlyser (Bedfont EC 50 Scientific), antes e depois do expediente de cada garçom.

**RESULTADOS:**

Os níveis pré-exposição de monóxido de carbono expirado foram semelhantes entre os garçons e os estudantes, mas após uma média de 9 horas no local de trabalho, o nível mediano entre os garçons foi mais que o dobro (de 2,0 ppm para 5,0 ppm, P < 0,001). Os níveis pós-exposição de monóxido de carbono correlacionaram com o número de mesas disponíveis para fumantes (Kendall's tau = 0,2, P < 0,0001).

**CONCLUSÃO:**

A exposição à fumaça de cigarro no ambiente de trabalho é a explicação mais provável para este aumento no nível de monóxido de carbono expirado entre os garçons. Estes achados podem ser utilizados para direcionar o debate sobre fumo passivo e a saúde pública.

## INTRODUCTION

Exposure to environmental tobacco smoke is a health risk that is of concern to patrons and of particular concern to employees of restaurants and bars. In a review of the literature, Siegel^1^ reported that levels of environmental tobacco smoke were 1.6 to 2.0 times higher than those found in other types of work-place and 1.5 times higher than those in homes with at least one smoker. Passive smoking has been found to increase the risk of developing lung cancer and ischemic heart disease.^2^ Consequently, cities in various countries have introduced laws that limit smoking to designated areas, but outright prohibition of smoking in restaurants has been less successful and has met opposition from restaurateurs and the tobacco industry.^3,4^ In 1995, the mayor of São Paulo passed a municipal decree completely banning smoking in restaurants. Prior to the implementation of the decree we undertook a study to measure levels of expired carbon monoxide in waiters, before and after a normal working day.

## METHODS

### Ethics

The procedures that follow were in accordance with the ethical standards of the committee responsible for human experimentation and with the Helsinki declaration of 1975, as revised in 1983.

### Setting

The survey was performed in São Paulo, Brazil, in July 1995 in a region near the center of the city. Fifteen restaurants with more than 50 tables (or at least 100 places) were approached. These restaurants were open for lunch and dinner: on average they served 50 meals at lunchtime and 30 meals in the evening. None of the restaurants had separated places for smokers and non-smokers. Seven restaurants refused to participate.

### Subjects

One hundred currently non-smoking waiters were contacted personally in the workplace and gave their consent to participate in the study. Smoking status was assessed by asking the subject if he was a current smoker. If he replied that he was not, he was asked if he had ever smoked, for how long, number of cigarettes smoked per day and the time, in months, since his last cigarette. No subject who had smoked a cigarette in the last month was included in the study.

### Main Measurements

Before starting work, each waiter was asked to respond to a short questionnaire that included questions on past smoking, exposure to cigarette smoke during the preceding 24 hours, and general questions about their health. Expired carbon monoxide level was measured using a desktop Smokerlyser (Bedfont EC 50 Scientific). In the restaurant, the measurement was performed in a place away from windows, doors, fans and air-conditioning vents. At the end of the working day the measurement was repeated. One hundred currently non-smoking medical students acted as a comparison group. They only provided one expired carbon monoxide measurement. Measurements were performed indoors, away from drafts and air currents.

**Table:** Levels of expired carbon monoxide in non-smoking waiters and medical students

Subjects	Median carbon monoxide - ppm (interquartile range)
Medical Students (n = 100)	2.5 (1.0 to 4.0)
Waiters: pre-exposure (n = 100)	2.0 (1.0 to 3.0)†
Waiters: post-exposure (n = 100)	5.0 (3.0 to 8.0)*

ppm = parts per million

† = Mann-Whitney test showing homogeneity of medical students’ and waiters’ pre-exposure carbon monoxide levels, P = 0.18

* = Wilcoxon test comparing waiters’ pre- and post-exposure carbon monoxide levels, P < 0.001.

### Statistical Methods

Non-parametric statistical tests (Wilcoxon and Mann-Whitney) were used to analyze the data because the carbon monoxide measurements were markedly and positively skewed. Data were not transformed due to the difficulty of handling the large number of zero values in the pre-exposure groups. The differences between pre- and post-exposure carbon monoxide levels were also positively skewed and included several zero values as well as a small number of negative values.

## RESULTS

The mean age of the waiters was 31.8 years (SD 9.6, range 18 to 54 years), 96% were men and they worked on average six days a week. Forty-two percent were ex-smokers who had quit a median of 2 years ago and 58% had never smoked. Ninety-four percent stated that their level of domestic exposure to cigarette smoke was zero, very low or low, and 67% said that exposure at work was moderate to very high. The medical students had a mean age of 21 years (SD 2.2, range 17 to 29 years), 56% were men, 66% had never smoked and 80% reported zero to low levels of domestic exposure to cigarette smoke. No subject had a pre-exposure carbon monoxide level greater than 8 ppm, which was considered to be within the normal range for non-smokers. Among the waiters, a repeat measurement of expired carbon monoxide levels was made after a mean of 9 hours (range 6 to 12 hrs) exposure at the workplace. Restaurants were by and large reasonably well ventilated, 37% had open windows, 72% open doors, 60% fans, 53% extractor fans in the kitchens and 8% air-conditioning.

Pre- and post-exposure carbon monoxide levels for the waiters are shown in Table. Following exposure at the workplace, the median carbon monoxide levels more than doubled. There was no correlation between reported levels of exposure in the work-place and post-exposure carbon monoxide levels (Kendall's tau = 0.07, P > 0.2) but a strong correlation between the number of tables available for smokers and post-exposure carbon monoxide levels was found (Kendall's tau = 0.2, P < 0.0001).

## DISCUSSION

Our results show that after a period of nine hours exposure to cigarette smoke in the work environment, median levels of expired carbon monoxide more than doubled among non-smoking waiters.

Reviewing the literature, we were unable to find other studies in which expired carbon monoxide had been measured before and after exposure in the work-place. Most studies either measure environmental levels of carbon monoxide, nicotine and particulate matter or saliva/serum cotinine levels. Jarvis et al^3^ performed a similar study among 42 non-smoking bar staff in London and Birmingham, UK, and found elevated post-exposure levels of saliva cotinine.

The elevated levels of expired carbon monoxide found among the waiters may not be entirely due to the inhalation of cigarette smoke, and other sources of carbon monoxide must be considered. Exposure to smoke from kitchens, barbecues fires or even from car exhaust fumes from outside the restaurant are all possibilities. However, the restaurants were well ventilated and most kitchens used hatches so that waiters did not have to enter them. Only two of the restaurants had barbecue facilities, which were fitted with exhaust flues. As already reported, 94% of the waiters stated that domestic exposure to cigarette smoke was zero, low or very low, a report that is substantiated by the low pre-exposure levels of carbon monoxide, which were comparable with those of the control group and within the normal range for non-smokers. We found a strong correlation between post-exposure expired carbon monoxide levels and the number of tables for smokers but not with the waiters’ own reports of environmental cigarette smoke exposure during the preceding 12 hours. Perhaps waiters exposed to high levels of cigarette smoke become accustomed to this level of exposure and no longer think it excessive. Whatever the explanation, the number of tables for smokers is probably a much more objective measure of environmental tobacco smoke exposure.

Provided that other sources of carbon monoxide exposure can be controlled, there are several advantages to measuring levels of expired carbon monoxide, rather than saliva or serum cotinine. The Smokerlyser is quick and easy to use, it is cheaper than performing a series of laboratory assays, and is more acceptable to the person giving the sample.

## CONCLUSION

Although many restaurants have both smoking and non-smoking areas, this division only provides some protection for the customer. Those who work in restaurants continue to be exposed to the harmful effects of tobacco smoke. If smoking is to be completely outlawed in restaurants, one factor that may promote such a change is non-smoking employees suing their employers for compensation because of the adverse health effects suffered as a consequence of occupational exposure to tobacco smoke. The case of Liesle Scholem in Australia shows that such cases can have a significant impact on smoke- free policies.^1,5^ Our results can be used both to inform the debate on passive smoking and as ammunition in the fight against smoking in public places.
